# The Expression of The Autophagy Gene Beclin-1 mRNA and Protein in Ectopic and Eutopic Endometrium of Patients with Endometriosis

**DOI:** 10.22074/ijfs.2015.4183

**Published:** 2015-02-07

**Authors:** Longyu Zhang, Ying Liu, Yuping Xu, Huan Wu, Zhaolian Wei, Yunxia Cao

**Affiliations:** 1 Reproductive Medicine Center, First Affiliated Hospital of Anhui Medical University, Hefei, China; 2 Anhui No.2 Provincial People’s Hospital, China

**Keywords:** Autophagy, Endometriosis, Beclin-1, CA125

## Abstract

**Background:**

To investigate the expression of Beclin-1 mRNA and protein in eutopic and ectopic endometrium of women with and without endometriosis, and evaluate the association of Beclin-1 protein expression and serum CA125 levels in the endometriosis group due to CA125 being a well-known biomarker of endometriosis.

**Materials and Methods:**

The expression levels (mean ± SD) of the mRNA and protein of Beclin-1 were examined in uterine endometria from 26 women without endometriosis and in eutopic and ectopic endometria from 26 endometriosis patients through experimental study, as reverse transcription PCR and Western-blotting assays. Serum CA125 levels in the endometriosis and control groups were compared and the correlation between Beclin-1 protein expression and serum CA125 was evaluated in the endometriosis group.

**Results:**

Both eutopic (0.12 ± 0.04, 1.25 ± 0.42) and ectopic (0.12 ± 0.05, 1.09 ± 0.50) endometriotic tissue from 26 women with endometriosis expressed significantly lower levels of Beclin-1 mRNA and protein than endometrium from 26 normal women (0.15 ± 0.02, 1.67 ± 0.44) (p<0.05). Serum CA125 levels were found to be significantly higher in the endometriosis group (p<0.05). In addition, Beclin-1 protein expression of eutopic endometria in patients with endometriosis was negatively correlated with serum CA125 (r= -0.57, p<0.01).

**Conclusion:**

The present study strongly suggests that Beclin-1 may play a role in the formation and progression of endometriosis.

## Introduction

Endometriosis is a common gynecological disease affecting 6 to 10% of women of reproductive age. Of these, 50 to 60% experience pelvic pain, and up to 50% of affected women experience infertility (1, 2). Dysmenorrhea and infertility are the main clinical manifestations of endometriosis. Endometriosis is also characterized by the presence of growing endometrial tissue outside of the uterine cavity, and by invasion, cultivation, and transfer. Infertility and chronic abdominal pain caused by endometriosis seriously impact the physical and mental health of women of reproductive age (3, 4). Ectopic endometria usually affect the ovaries and can cause ovarian endometriosis (OEM). Repeated cyclic hemorrhage can destroy ovarian tissue, and cause inflammation, pelvic tissue adhesion and distorsion of pelvic anatomy.

Autophagy is a form of programmed cell death, distinct from apoptosis, that has special morphological changes and a unique regulatory pathway. For example, the autophagic body is not dependent on cysteine-aspartic protease (caspase). Autophagy plays a very important role in the process of cell growth, differentiation, tissue remodeling, cell immunity, environmental adaptation and death. The features of autophagic programmed cell death include double-layered autophagosome and multilayered membrane-bound bubble structures that encase massive amounts of cytoplasm and a number of organelles. The plasmin system eventually removes these components (5, 6).

Beclin-1, the mammalian homologous gene of yeast Atg6, has a central role in autophagy (7). The expression of the Beclin-1 protein was found to be very low in a variety of tumor cells. Liang et al. found that the level of Beclin-1 in breast carcinoma was significantly lower than in normal breast epithelial tissues; the transfection of Beclin-l to human breast cancer cell line MCF7 inhibited *in vitro* proliferation and tumorigenicity of these cells, suggesting that the decline of cell autophagic activity may be associated with the initiation and development of tumors (8). Ectopic endometrial cells from endometriosis patients have similar biological characteristics to those of cancer cells. These include invasion, implantation and transfer. Also, both endometriosis and cancer patients are subject to relapse. Both endometriosis and adenomyosis are characterized by the presence of ectopic endometria outside the uterus and in the myometrial wall. Recent morphologic studies have demonstrated that in both endometriosis and adenomyosis, the eutopic endometrium and the inner myometrium show functional and structural abnormalities (9). Studies have also shown that Beclin-1 mRNA and protein expression are significantly lower in the eutopic endometrium of women with adenomyosis than in healthy women. Furthermore, Beclin- 1 has been found to be negatively correlated with serum cancer antigen 125 (CA125) and pelvic pain in patients with adenomyosis (10). However, no studies on the expression of Beclin-1 in endometriosis have yet been published.

CA125 is a cell surface antigen and a member of the mucin family of glycoproteins, encoded by MUC-16. It was discovered in the early 1980s. Immunocytochemical-based studies have demonstrated the presence of CA125 on the surface of cells in endometriotic lesions (11). CA125 is a well-known biomarker of endometriosis and can be helpful in daily clinical practice when endometriosis is suspected (12).

The purpose of the present study is to compare the expression levels of Beclin-1 mRNA and protein in uterine endometria of women without endometriosis to those in eutopic and ectopic endometria of endometriosis patients using Real time-PCR and Western blot analysis, and to evaluate the association between Beclin-1 protein expression and serum CA125 levels in the endometriosis group.

## Materials and Methods

### Subjects

The experimental study was approved by the Institute Research Medical Ethics Committee of Anhui Medical University and written informed consent for participation in the study was provided by each participant. A total of 52 patients were selected using the following inclusion criteria: reproductive age (18- 40 years). Proliferative phase of menstrual cycle,no medical treatments during 3 months before operation and no uterine devices. All patients were evaluated in days 3-7 after menstruation. The patients were divided into an endometriosis group and a normal endometrial tissue group. The endometriosis group (experimental group) comprised of patients who had unilateral or bilateral ovarian chocolate cysts with diameters ≥3 cm. 26 samples of eutopic endometrium and ectopic ovarian endometrial tissue were collected from patients undergoing laparoscopy or laparotomy with chocolate cyst tissue confirmed by pathology. The proliferative phase was confirmed by pathological diagnosis. Normal endometrial tissue group (control group) consisted of endometrium samples collected from 26 sterile women with menstrual regularity whose infertility was attributed to tubal factor. Endometriosis was excluded by laparoscopy. Samples were collected during laparoscopy or pelvic exam and confirmed by pathological examination. Endometrial cell sampler (FEM103000, Unimax medical systems Inc., Taiwan) was used to draw the eutopic proliferative endometria from endometriosis patients and the controls.

Patient information including age, body mass index (BMI), serum CA125 level, previous pregnancy rate (number of previously pregnant women divided by 26) and previous dilatation and curettage rate (number of women who underwent dilatation and curettage divided by 26) was collected from the patients’ clinical records and laboratory examination (Table 1).

**Table 1:** Comparison of demographic and Beclin-1 expression data between endometriosis and control groups


Parameter	Endometriosis	Control	P value

**Age (Y)**	34.28 ± 4.33	32.12± 4.42	0.087
**BMI (kg/m^2^)**	21.30 ± 2.01	20.80± 2.20	0.418
**CA125 level (U/mL)**	43.17 ± 52.24	12.01± 1.82	0.004
**Previous pregnancy rate**	19.23%	7.68%	0.223
**Previous dilatation and curettage rate**	13.04%	3.85%	0.298
**Number of RT-PCR**	26	26	
**Number of Western blot**	26	26	
**mRNA expression of Beclin-1 in endometrial tissue (ectopic)**	0.115 ± 0.046	0.152± 0.022	0.001
**mRNA expression of Beclin-1 in endometrial tissue (eutopic)**	0.118 ± 0.042	0.152± 0.022	0.002
**Protein expression of Beclin-1 in endometrial tissue (ectopic)**	1.091 ± 0.499	1.669± 0.439	<0.001
**Protein expression of Beclin-1 in endometrial tissue (eutopic)**	1.252 ± 0.424	1.669 ± 0.439	0.001


Patient demographic data including age, body mass index (BMI), previous pregnancy rate and previous dilatation and curettage rate showed no obvious difference. Serum CA125 level was elevated in the endometriosis group. The normalized expression level of Beclin-1 mRNA in the experimental group was significantly lower than in the control group (p<0.05). The expression level of Beclin-1 protein in the experimental group was significantly lower than in the control group (p<0.05).

### Immunoassay for CA-125 determination

Blood samples for CA125 were taken prior to surgery, centrifuged and assayed in accordance with the manufacturer’s instructions. Electrochemiluminescent immunoassay was used to determine CA125 serum levels in both groups. The CA125 kit and the automatic immunoassay analyzer were manufactured by Abbott Laboratories, USA. Normal levels of CA125 were considered less than 35 U/mL.

### Reverse transcription-polymerase chain reaction (RT-PCR)

#### RNase exclusion

The grinding device was cleaned using standard methods and rinsed four times with sterilized double- distilled water. High-pressure disinfection was used to remove diethypyrocarbonate (DEPC). The device was then dried for 1 hour at 200˚C. After DEPC was added to the solution to a concentration of 0.1% and the mixture was subjected to magnetic stirring for 20 minutes at room temperature. It was then heated to 70˚C for 1 hour to remove residual DEPC. The plastic products were soaked in 0.1% DEPC overnight, dried, and autoclaved for 15-30 minutes.

#### Total RNA extraction and cDNA synthesis

Total RNA was extracted (100 mg of tissue) using TRIzol reagent (Invitrogen Life Technologies, Paisley, U.K.) according to the manufacturer’s instructions. RNA was stored at -80˚C for future procedures.

According to the manufacturer’s instructions, cDNA was synthesized using AMV Reverse Transcriptase (Promega Biotech, Beijing, China) starting with 1 μg of mRNA.

### Amplification of cDNA

This process began with a 5-minute denaturation at 94˚C followed by 30 cycles of 30 seconds of denaturation at 94˚C, 30 seconds of annealing at 57˚C and 30 seconds of extension at 72˚C. This was followed by a 10-minute extension at 72˚C.

#### Western blot analysis

Protein samples were separated on a 1% agarose gel under denaturing conditions and electrophoretically transferred to nitrocellulose membranes. The membranes were probed with 1:500 dilution of Beclin-1antibody (sc-11427, Santa Cruz Biotechnology, Inc., Santa Cruz, CA, U.S.) and then incubated with a secondary antibody (anti-mouse IgG, 1:5000; Beyotime Biotech, Shanghai, China). For detection, enhanced chemiluminescence was carried out with BCA kits (Beyotime Biotech, Shanghai, China). An internal reference sample (the same one for each blot) was included as a standard for quantification. The signal from each band was measured against the standard, and this relative number was used for statistical analysis.

### Statistical analysis

Statistical analysis was performed using SPSS 19.0 statistical software package (SPSS Inc., Chicago, IL). Data were expressed as mean ± standard deviation (SD) or percentages as appropriate. A single-sample Kolmogorov-Simirnov test was used to test normality and an independent t test was used for quantitative variables. The ÷2 test was used for qualitative variables. ANOVA was used for comparing multiple groups and the Q test was used to contrast between two groups. P<0.05 was considered statistically significant.

## Results

The mean ages of women with and without endometriosis were 34.28 ± 4.33 and 32.12 ± 4.42 years respectively with no significant difference between them (p=0.09). There was no difference in BMI, number of previous pregnancy rate and previous dilatation curettage rate. Serum levels of CA125 were significantly higher in the endometriosis group than in the control group (Table 1).

### Expression of Beclin-1 mRNA in eutopic and ectopic endometrial tissue

**Fig 1:**
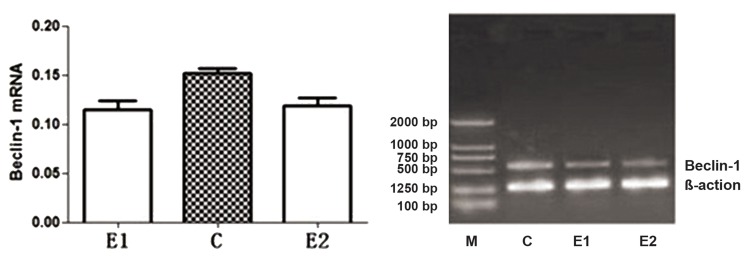
Semi-quantitative expression of Beclin-1 mRNA in the experimental group (paired eutopic and ectopic endometrial samples from the same patients) and the samples from the control group was detected using RT-PCR. The grey value was analyzed using the Jetta 801 gel imaging software. E1; Endometriosis ectopic group, C; Control group and E2; Endometriosis eutopic group.

Semi-quantitative expression of Beclin-1 mRNA in the experimental group samples (paired eutopic and ectopic endometrial samples from the same patients) and the samples from the control group was detected using RT-PCR. The normalized expression level of Beclin-1 mRNA in the experimental group was significantly lower than in the control group (p<0.05) (Table 1, Fig 1). The grey value was analyzed using Jetta 801 gel imaging software.

### Expression level of Beclin-1 protein in eutopic and ectopic endometrial tissue

**Fig 2:**
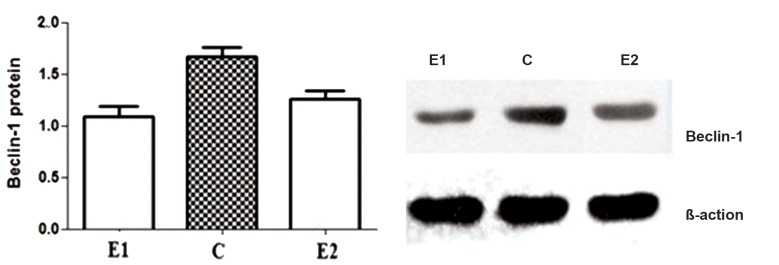
Beclin-1 protein expression presented with representative immunoblots. The protein bands of β-actin in these groups were consistent. E1; Endometriosis ectopic group, C; Control group and E2; Endometriosis eutopic group.

The expression of Beclin-1 protein was analyzed in the experimental (in eutopic and ectopic endometrium group) and control groups by Western blotting. The expression level of Beclin-1 protein in the experimental group was significantly lower than in the control group (p<0.05) (Table 1, Fig 2). The protein bands of β-actin in these groups were obvious and consistent. Difference in Beclin-l protein levels was observed among the three groups of endometrial tissue.

### The correlation between the levels of Beclin-1 protein expression in endometria and serum levels of CA125

**Fig 3:**
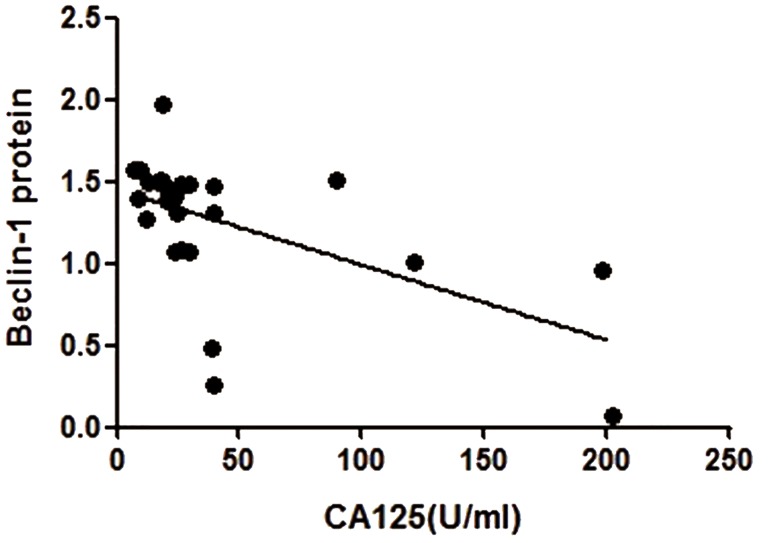
There was a negative correlation between Beclin-1 protein expression and serum CA125 (n=26, r=-0.566, p=0.003). The line refers to the changing trend of data.

Serum levels of CA125 ranged from 6.98 to 201.7 U/ ml. Pearson’s correlation coefficient (r) of CA125 level and Beclin-1 protein expression level in eutopic endometria of the experimental group was -0.566. This negative correlation between the serum CA125 level and Beclin-1 protein expression level was statistically significant (p=0.003) (Fig 3).

## Discussion

This study is the first to reveal that the expression of Beclin-1 mRNA and protein is significantly decreased in ectopic and eutopic endometrium from patients with endometriosis. In contrast, serum CA125 levels were significantly higher in the endometriosis group. Beclin-1 protein expression of eutopic endometria in patients with endometriosis was negatively correlated with serum CA125 level. Since Beclin-1 is an autophagy-related protein, it was deduced that the ability of cells to undergo autophagy was reduced in ectopic and eutopic endometria of patients with endometriosis, and that autophagy might be related to the pathogenesis and progression of endometriosis.

Theories concerning the etiology of endometriosis include the implantation hypothesis of retrograde menstruation proposed by Sampson in 1927, the doctrine of hematogenous and lymphatic dissemination, coelomic epithelium metaplasia theory, immune theory and the theory of heredity and environment. Studies have shown that endometrial fragments (glandular epithelial and stromal cells) must undergo the adhesion, invasion and angiogenesis process so that they can grow, and cause lesions and clinical symptoms. However, this hypothesis cannot completely explain why retrograde menstruation does not causeendometriosis in all women, indicating that other factors may play important roles in the pathogenesis of endometriosis. Eutopic endometria may play a role in the initiation and progression of endometriosis and it is thought to be the source of lesions (13). The so-called eutopic endometrium determinism was used to revise and modify Sampson’s hypothesis. Although the eutopic endometria of women with and without endometriosis are histologically similar, studies have shown that invasive properties, decreased apoptosis, alterations in expression of specific genes and proteins, and increased steroid and cytokine production have been observed in the eutopic endometria of women with endometriosis. Also,significant biochemical differences exist even between ectopic and autologous eutopic endometria (14).

The Beclin-1 gene is essential to autophagy. Beclin- 1 is located on human chromosome 17q21. It is 150 kb in length, and is one of about 12 genes in the 400 kb region (15). Liang et al. found the structure of Beclin-1 to be similar to that of the yeast autophagy gene apg6/ vps30 and that Beclin- 1 is part of the Bcl-2 protein family (8). One study on sequence and structure of this gene indicated that Beclin-1 contains a conserved BH3 domain, which is both necessary and sufficient for its interaction with Bcl-X (L) (16). The present work showed that expression of Beclin-1 mRNA and protein was lower in both eutopic and ectopic endometrium of patients with endometriosis than in the control group. Decreased expression of Beclin- 1 has been found to be inversely correlated with altered expression of Bcl-xL in ovarian carcinoma, and its expression has thus been used to predict patient survival in ovarian carcinomas with increased expression of Bcl-xL (17). Sun et al. reported that Beclin-1 protein was up-regulated in overexpressed transfectants of CaSki cells in cervical cancer (18). Other studies have shown the possibility that members of the Bcl-2 family may function as oncogenes not only by blocking apoptosis but also by blocking autophagy (19).

Tumors were one of the first conditions to be found related to autophagy. It has been confirmed that autophagy is related to precancerous lesions involving carcinoma cells. Removal of Beclin-1 gene from mouse embryonic stem cells showed that beclin-1-/- mutant mice die early during embryogenesis and that beclin-1+/- mutant mice suffer from a high incidence of spontaneous tumors (20). Beclin-1 is deleted in 40- 75% of cases of human sporadic breast, ovarian, and prostate cancers. Knocking down ATG5 and beclin-1, two known essential autophagic molecules, can cause radiation resistance among lung cancer cells. Therefore, reinforcement of autophagy may improve the effects of radiation therapy on lung cancer cells (21).

Results showed that the decreased autophagic activity observed in ectopic and eutopic endometrial cells can lead to less autophagy-dependent degradation of proteins and less programmed cell death. In this way, ectopic endometrial cells can adhere to the extracellular matrix, invade other tissues, grow outside the endometrium and thus cause endometriosis. The correlation between levels of expression and the concentration of interactive sites between Beclin-1 and members of the Bcl-2 family suggest that Beclin-1 may be involved in apoptotic processes that also involve the Bcl-2 family. The inactivation of Beclin-1 results in loss of control of certain genes in the Bcl-2 family and can decrease capacity for autophagy in cells, allowing ectopic endometrial cells to survive.

The result of the present study showed that concentrations of CA 125 were higher in patients with endometriosis than in the control group. These results are similar to those found by Ramos et al. (22). CA 125 is expressed in the eutopic and ectopic endometrium. The special function of oligosaccharides linked to CA125 may induce immunomodulatory effects and CA 125 may potently suppress the cytotoxicity of human natural killer (NK) cells (23, 24), thereby promoting progression of the disease. Serum CA 125 levels are non-surgical tools for diagnosing and staging pelvic endometriosis, and the value of serum CA 125 measurement as a diagnostic aid in moderate-severe stages is well recognized (25). Maiorana et al. showed that the mean serum CA 125 levels were higher during stage IV than during other stages of endometriosis according to criteria issued by the American Fertility Society (R-AFS) (26).

## Conclusion

Results suggest that Beclin-1 is associated with the occurrence and development of endometriosis and is negatively correlated with CA125 serum level. These provide novel insight into the pathogenesis of endometriosis. However, the present experiment was not sufficiently thorough. Further experiments on populations with larger sample may further substantiate the association between autophagy and endometriosis. Further investigation should focus on the relationship between the expression of Beclin-l in endometrial tissues and clinical symptoms.

## References

[1] Eskenazi B, Warner ML (1997). Epidemiology of endometriosis. Obstet Gynecol Clin North Am.

[2] Goldstein DP, deCholnoky C, Emans SJ, Leventhal JM (1980). Laparoscopy in the diagnosis and management of pelvic pain in adolescents. J Reprod Med.

[3] Frackiewicz EJ (2000). Endometriosis: an overview of the disease and its treatment. J Am Pharm Assoc (Wash).

[4] Yang MH, Wang PH, Wang SJ, Sun WZ, Oyang YJ, Fuh JL (2012). Women with endometriosis are more likely to suffer from migraines: a population-based study. PLoS One.

[5] Klionsky DJ (2005). Autophagy. Curr Biol.

[6] Lockshin RA, Zakeri Z (2004). Apoptosis, autophagy, and more. Int J Biochem Cell Biol.

[7] Kang R, Zeh HJ, Lotze MT, Tang D (2001). The Beclin 1 network regulates autophagy and apoptosis. Cell Death Differ.

[8] Liang XH, Jackson S, Seaman M, Brown K, Kempkes B, Hibshoosh H (1999). Induction of autophagy and inhibition of tumorigenesis by Beclin-1. Nature.

[9] Brosens I, Kunz G, Benagiano G (2012). Is adenomyosis the neglected phenotype of an endomyometrial dysfunction syndrome?. Gynecol Surg.

[10] Ren Y, Mu L, Ding X, Zheng W (2010). Decreased expression of Beclin 1 in eutopic endometrium of women with adenomyosis. Arch Gynecol Obstet.

[11] Barbieri RL, Niloff JM, Bast RC Jr, Scaetzl E, Kistner RW, Knapp RC (1986). Elevated serum concentrations of CA-125 in patients with advanced endometriosis. Fertil Steril.

[12] Szubert M, Suzin J, Wierzbowski T, Kowalczyk-Amico K (2012). CA-125 concentration in serum and peritoneal fluid in patients with endometriosis - preliminary results. Arch Med Sci.

[13] Rai P, Kota V, Deendayal M, Shivaji S (2010). Differential proteome profiling of eutopic endometrium from women with endometriosis to understand etiology of endometriosis. J Proteome Res.

[14] Ulukus M, Cakmak H, Arici A (2006). The role of endometrium in endometriosis. J Soc Gynecol Investig.

[15] Aita VM, Liang XH, Murty VV, Pincus DL, Yu W, Cayanis E (1999). Cloning and genomic organization of beclin 1, a candidate tumor suppressor gene on chromosome 17q21. Genomics.

[16] Oberstein A, Jeffrey PD, Shi Y (2007). Crystal structure of the Bcl-XL-Beclin l peptide complex: Beclin l is a novel BH3-only protein. J Biol Chem.

[17] Lin HX, Qiu HJ, Zeng F, Rao HL, Yang GF, Kung HF (2013). Decreased expression of Beclin 1 correlates closely with Bcl-xL expression and poor prognosis of ovarian carcinoma. PLoS One.

[18] Sun Y, Liu JH, Jin L, Pan L, Sui YX, Yang Y (2012). Beclin 1 influences cisplatin-induced apoptosis in cervical cancer CaSki cells by mitochondrial dependent pathway. Int J Gynecol Cancer.

[19] Pattingre S, Levine B (2006). Bcl-2 inhibition of autophagy: a new route to cancer?. Cancer Res.

[20] Yue Z, Jin S, Yang C, Levine AJ, Heintz N (2003). Beclin 1, an autophagy gene essential for early embryonic development, is a haploinsufficient tumor suppressor. Proc Natl Acad Sci USA.

[21] Kim KW, Hwang M, Moretti L, Jaboin JJ, Cha YI, Lu B (2008). Autophagy upregulation by inhibitors of caspase-3 and mTOR enhances radiotherapy in a mouse model of lung cancer. Autophagy.

[22] Ramos IM, Podgaec S, Abrao MS, Oliveira Rd, Baracat EC (2012). Evaluation of CA-125 and soluble CD-23 in patients with pelvic endometriosis: a case-control study. Rev Assoc Med Bras.

[23] Kui Wong N, Easton RL, Panico M, Sutton-Smith M, Morrison JC, Lattanzio FA (2003). Characterization of the oli gosaccharides associated with the human ovarian tumor marker CA125. J Biol Chem.

[24] Patankar MS, Jing Y, Morrison JC, Belisle JA, Lattanzio FA, Deng Y (2005). Potent suppression of natural killer cell response mediated by the ovarian tumor marker CA125. Gynecol Oncol.

[25] Mihalyi A, Gevaert O, Kyama CM, Simsa P, Pochet N, De Smet F (2010). Non-invasive diagnosis of endometriosis based on a combined analysis of six plasma biomarkers. Hum Reprod.

[26] Maiorana A, Cicerone C, Niceta M, Alio L (2007). Evaluation of serum CA 125 levels in patients with pelvic pain related to endometriosis. Int J Biol Markers.

